# HydDB: A web tool for hydrogenase classification and analysis

**DOI:** 10.1038/srep34212

**Published:** 2016-09-27

**Authors:** Dan Søndergaard, Christian N. S. Pedersen, Chris Greening

**Affiliations:** 1 Aarhus University, Bioinformatics Research Centre, C.F. Møllers Allé 8, Aarhus DK-8000, Denmark; 2 The Commonwealth Scientific and Industrial Research Organisation, Land and Water Flagship, Clunies Ross Street, Acton, ACT 2601, Australia; 3 Monash University, School of Biological Sciences, Clayton, VIC 3800, Australia

## Abstract

H_2_ metabolism is proposed to be the most ancient and diverse mechanism of energy-conservation. The metalloenzymes mediating this metabolism, hydrogenases, are encoded by over 60 microbial phyla and are present in all major ecosystems. We developed a classification system and web tool, HydDB, for the structural and functional analysis of these enzymes. We show that hydrogenase function can be predicted by primary sequence alone using an expanded classification scheme (comprising 29 [NiFe], 8 [FeFe], and 1 [Fe] hydrogenase classes) that defines 11 new classes with distinct biological functions. Using this scheme, we built a web tool that rapidly and reliably classifies hydrogenase primary sequences using a combination of *k*-nearest neighbors’ algorithms and CDD referencing. Demonstrating its capacity, the tool reliably predicted hydrogenase content and function in 12 newly-sequenced bacteria, archaea, and eukaryotes. HydDB provides the capacity to browse the amino acid sequences of 3248 annotated hydrogenase catalytic subunits and also contains a detailed repository of physiological, biochemical, and structural information about the 38 hydrogenase classes defined here. The database and classifier are freely and publicly available at http://services.birc.au.dk/hyddb/

Microorganisms conserve energy by metabolizing H_2_. Oxidation of this high-energy fuel yields electrons that can be used for respiration and carbon-fixation. This diffusible gas is also produced in diverse fermentation and anaerobic respiratory processes^1^. H_2_ metabolism contributes to the growth and survival of microorganisms across the three domains of life, including chemotrophs and phototrophs, lithotrophs and heterotrophs, aerobes and anaerobes, mesophiles and extremophiles alike^1^,^2^. On the ecosystem scale, H_2_ supports microbial communities in most terrestrial, aquatic, and host-associated ecosystems^1^,^3^. It is also proposed that H_2_ was the primordial electron donor^4^,^5^. In biological systems, metalloenzymes known as hydrogenases are responsible for oxidizing and evolving H_2_^1^,^6^. Our recent survey showed there is a far greater number and diversity of hydrogenases than previously thought^2^. It is predicted that over 55 microbial phyla and over a third of all microorganisms harbor hydrogenases^2^,^7^. Better understanding H_2_ metabolism and the enzymes that mediate it also has wider implications, particularly in relation to human health and disease^3^,^8^, biogeochemical cycling^9^, and renewable energy^10^,^11^.

There are three types of hydrogenase, the [NiFe], [FeFe], and [Fe] hydrogenases, that are distinguished by their metal composition. Whereas the [Fe]-hydrogenases are a small methanogenic-specific family^12^, the [NiFe] and [FeFe] classes are widely distributed and functionally diverse. They can be classified through a hierarchical system into different groups and subgroups/subtypes with distinct biochemical features (e.g. directionality, affinity, redox partners, and localization) and physiological roles (i.e. respiration, fermentation, bifurcation, sensing)^1^,^6^. It is necessary to define the subgroup or subtype of the hydrogenase to predict hydrogenase function. For example, while Group 2a and 2b [NiFe]-hydrogenases share >35% sequence identity, they have distinct roles as respiratory uptake hydrogenases and H_2_ sensors respectively^13^,^14^. Likewise, discrimination between Group A1 and Group A3 [FeFe]-hydrogenases is necessary to distinguish fermentative and bifurcating enzymes^2^,^15^. Building on previous work^16^,^17^, we recently created a comprehensive hydrogenase classification scheme predictive of biological function^2^. This scheme was primarily based on the topology of phylogenetic trees built from the amino acid sequences of hydrogenase catalytic subunits/domains. It also factored in genetic organization, metal-binding motifs, and functional information. This analysis identified 22 subgroups (within four groups) of [NiFe]-hydrogenases and six subtypes (within three groups) of [FeFe]-hydrogenases, each proposed to have unique physiological roles and contexts^2^.

In this work, we build on these findings to develop the first web database for the classification and analysis of hydrogenases. We developed an expanded classification scheme that captures the full sequence diversity of hydrogenase enzymes and predicts their biological function. Using this information, we developed a classification tool based on the *k*-nearest neighbors’ (*k*-NN) method. HydDB is a user-friendly, high-throughput, and functionally-predictive tool for hydrogenase classification that operates with precision exceeding 99.8%.

## Results and Discussion

### A sequence-based classification scheme for hydrogenases

**Figure 1:**
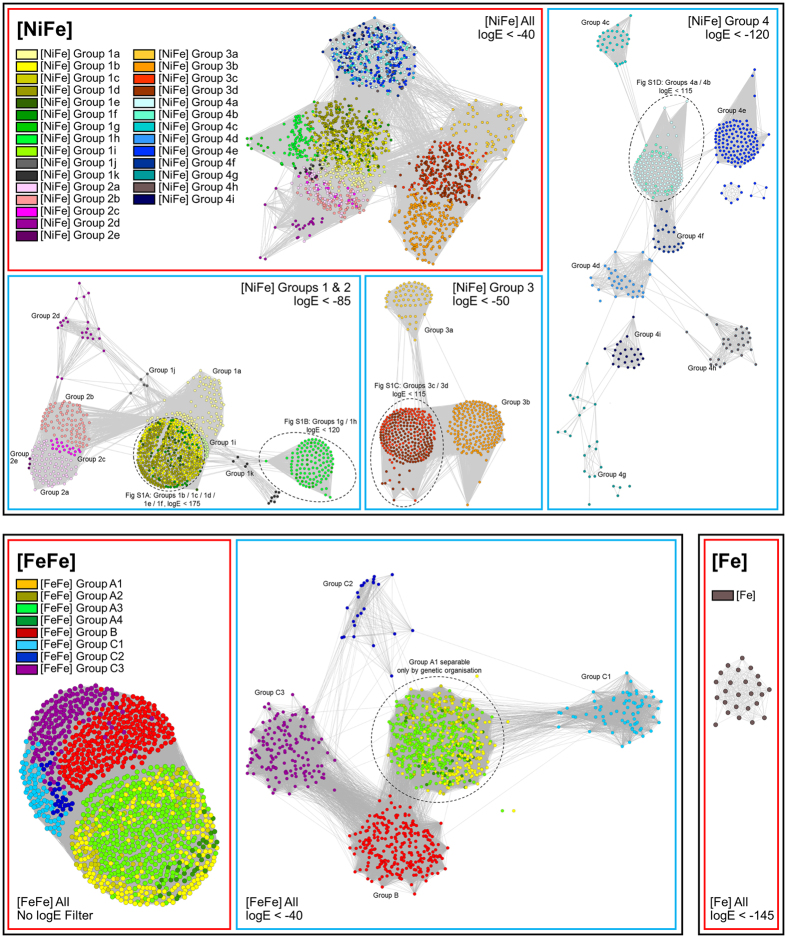
Sequence similarity network of hydrogenase sequences. Nodes represent individual proteins and the edges show the BLAST *E*-values between them at the log*E* filter defined at the bottom-left of each panel. The sequences are colored by class as defined in the legends. Figure S1 shows the further delineation of the encircled [NiFe] hydrogenase classes.

We initially developed a classification scheme to enable prediction of hydrogenase function by primary sequence alone. To do this, we visualized the relationships between all hydrogenases in sequence similarity networks (SSN)^18^, in which nodes represent individual proteins and the distances between them reflect BLAST *E*-values. As reflected by our analysis of other protein superfamilies^19^,^20^, SSNs allow robust inference of sequence-structure-function relationships for large datasets without the problems associated with phylogenetic trees (e.g. long-branch attraction). Consistent with previous phylogenetic analyses^2^,^16^,^17^, this analysis showed the hydrogenase sequences clustered into eight major groups (Groups 1 to 4 [NiFe]-hydrogenases, Groups A to C [FeFe]-hydrogenases, [Fe]-hydrogenases), six of which separate into multiple functionally-distinct subgroups or subtypes at narrower log*E* filters (Fig. 1; Figure S1). The SSNs demonstrated that all [NiFe]-hydrogenase subgroups defined through phylogenetic trees in our previous work^2^ separated into distinct clusters, which is consistent with our evolutionary model that such hydrogenases diverged from a common ancestor to adopt multiple distinct functions^2^. The only exception were the Group A [FeFe]-hydrogenases, which, as previously-reported^2^,^17^, cannot be classified by sequence alone as they have principally diversified through changes in domain architecture and quaternary structure. It remains necessary to analyze the organization of the genes encoding these enzymes to determine their specific function, e.g. whether they serve fermentative or electron-bifurcating roles.

The SSN analysis revealed that several branches that clustered together on the phylogenetic tree analysis^2^ in fact separate into several well-resolved subclades (Fig. 1). We determined whether this was significant by analyzing the taxonomic distribution, genetic organization, metal-binding sites, and reported biochemical or functional characteristics of the differentiated subclades. On this basis, we concluded that 11 of the new subclades identified are likely to have unique physiological roles. We therefore refine and expand the hydrogenase classification to reflect the hydrogenases are more diverse in both primary sequence and predicted function than accounted for by even the latest classification scheme^2^. The new scheme comprises 38 hydrogenase classes, namely 29 [NiFe]-hydrogenase subclasses, 8 [FeFe]-hydrogenase subtypes, and the monophyletic [Fe]-hydrogenases (Table 1).

Three lineages originally classified as Group 1a [NiFe]-hydrogenases were reclassified as new subgroups, namely those affiliated with Coriobacteria (Group 1i), Archaeoglobi (Group 1j), and Methanosarcinales (Group 1i). Cellular and molecular studies show these enzymes all support anaerobic respiration of H_2_, but differ in the membrane carriers (methanophenazine, menaquinone) and terminal electron acceptors (heterodisulfide, sulfate, nitrate) that they couple to^21^,^22^. The previously-proposed 4b and 4d subgroups^2^ were dissolved, as the SSN analysis confirmed they were polyphyletic. These sequences are reclassified here into five new subgroups: the formate- and carbon monoxide-respiring Mrp-linked complexes (Group 4b)^23^, the ferredoxin-coupled Mrp-linked complexes (Group 4d)^24^, the well-described methanogenic Eha (Group 4h) and Ehb (Group 4i) supercomplexes^25^, and a more loosely clustered class of unknown function (Group 4g). Enzymes within these subgroups, with the exception of the uncharacterized 4g enzymes, sustain well-described specialist functions in the energetics of various archaea^23^,^24^,^25^. Three crenarchaeotal hydrogenases were also classified as their own family (Group 2e); these enzymes enable certain crenarchaeotes to grow aerobically on O_2_^26^,^27^ and hence may represent a unique lineage of aerobic uptake hydrogenases currently underrepresented in genome databases. The Group C [FeFe]-hydrogenases were also separated into three main subtypes given they separate into distinct clusters even at relatively broad log*E* values (Fig. 1); these subtypes are each transcribed with different regulatory elements and are likely to have distinct regulatory roles^2^,^17^,^28^ (Table 1).

### HydDB reliably predicts hydrogenase class using the *k*-NN method and CDD referencing

Using this information, we built a web tool to classify hydrogenases. Hydrogenase classification is determined through a three-step process following input of the catalytic subunit sequence. Two checks are initially performed to confirm if the inputted sequence is likely to encode a hydrogenase catalytic subunit/domain. The Conserved Domain Database (CDD)^29^ is referenced to confirm that the inputted sequence has a hydrogenase catalytic domain, i.e. “Complex1_49kDa superfamily” (cl21493) (for NiFe-hydrogenases), “Fe_hyd_lg_C superfamily” (cl14953) (for FeFe-hydrogenases), and “HMD” (pfam03201) (for Fe-hydrogenases). A homology check is also performed that computes the BLAST *E*-value between the inputted sequence and its closest homolog in HydDB. HydDB classifies any inputted sequence that lacks hydrogenase conserved domains or has low homology scores (*E*-value > 10^−5^) as a non-hydrogenase (Table S1).

**Figure 2:**
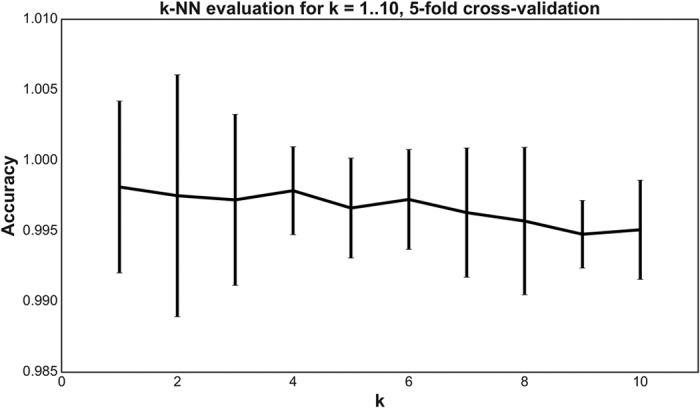
Evaluating the *k*-NN classifier for *k* = 1…10. For each *k*, a 5-fold cross-validation was performed. The mean precision ± two standard deviations of the folds is shown in the figure (note the y-axis). *k* = 1 provides the most accurate classifier. However, *k* = 4 provides almost the same precision and is more robust to errors in the training set (reflected by the lower standard deviation). In general, the standard deviation is very small, indicating that the predictions are robust to changes in the training data.

In the final step, the sequence is classified through the *k*-NN method that determines the most similar sequences listed in the HydDB reference database. To determine the optimal *k* for the dataset, we performed a 5-fold cross-validation for *k* = 1…10 and computed the precision for each *k*. The results are shown in Fig. 2. The classifier predicted the classes of the 3248 hydrogenase sequences with 99.8% precision and high robustness when performing a 5-fold cross-validation (as described in the Methods section) for *k* = 4. The six sequences where there were discrepancies between the SSN and *k*-NN predictions are shown in Table S2. The classifier has also been trained to detect and exclude protein families that are homologous to hydrogenases but do not metabolize H_2_ (Nuo, Ehr, NARF, HmdII^1^,^2^) using reference sequences of these proteins (Table S1).

Sequences of the [FeFe] Group A can be classified into functionally-distinct subtypes (A1, A2, A3, A4) based on genetic organization^2^. The classifier can classify such hydrogenases if the protein sequence immediately downstream from the catalytic subunit sequence is provided. The classifier references the CDD to search for conserved domains in the downstream protein sequence. A sequence is classified as [FeFe] Group A2 if one of the domains “GltA”, “GltD”, “glutamate synthase small subunit” or “putative oxidoreductase”, but not “NuoF”, is found in the sequence. Sequences are classified as [FeFe] Group A3 if the domain “NuoF” is found and [FeFe] Group A4 if the domain “HycB” is present. If none of the domains are found, the sequence is classified as A1. These classification rules were determined by collecting 69 downstream protein sequences. The sequences were then submitted to the CDD and the domains which most often occurred in each subtype were extracted.

In addition to its precision, the classifier is superior to other approaches due to its usability. It is accessible as a free web service at http://services.birc.au.dk/hyddb/ HydDB allows the users to paste or upload sequences of hydrogenase catalytic subunit sequences in FASTA format and run the classification (Figure S2). When analysis has completed, results are presented in a table that can be downloaded as a CSV file (Figure S3). This provides an efficient and user-friendly way to classify hydrogenases, in contrast to the previous standard which requires visualization of phylogenetic trees derived from multiple sequence alignments^30^.

### HydDB infers the physiological roles of H_2_ metabolism

As summarized in Table 1, hydrogenase class is strongly correlated with physiological role. As a result, the classifier is capable of predicting both the class and function of a sequenced hydrogenase. To demonstrate this capacity, we used HydDB to analyze the hydrogenases present in 12 newly-sequenced bacteria, archaea, and eukaryotes of major ecological significance. The classifier correctly classified all 24 hydrogenases identified in the sequenced genomes, as validated with SSNs (Table 2). On the basis of these classifications, the physiological roles of H_2_ metabolism were predicted (Table 2). For five of the organisms, these predictions are confirmed or supported by previously published data^27^,^31^,^32^,^33^,^34^. Other predictions are in line with metabolic models derived from metagenome surveying^35^,^36^,^37^. In some cases, the capacity for organisms to metabolize H_2_ was not tested or inferred in previous studies despite the presence of hydrogenases in the sequenced genomes^32^,^38^,^39^,^40^.

While HydDB serves as a reliable initial predictor of hydrogenase class and function, further analysis is recommended to verify predictions. Hydrogenase sequences only provide organisms with the genetic capacity to metabolise H_2_; their function is ultimately modulated by their expression and integration within the cell^1^,^41^. In addition, some classifications are likely to be overgeneralized due to lack of functional and biochemical characterization of certain lineages and sublineages. For example, it is not clear if two distant members of the Group 1h [NiFe]-hydrogenases (*Robiginitalea biformata*, *Sulfolobus islandicus*) perform the same H_2_-scavenging functions as the core group^9^. Likewise, it seems probable that the Group 3a [NiFe]-hydrogenases of Thermococci and Aquificae use a distinct electron donor to the main class^42^. Prominent cautions are included in the enzyme pages in cases such as these. HydDB will be updated when literature is published that influences functional assignments.

### HydDB contains interfaces for hydrogenase browsing and analyzing

In addition to its classification function, HydDB is designed to be a definitive repository for hydrogenase retrieval and analysis. The database presently contains entries for 3248 hydrogenases, including their NCBI accession numbers, amino acid sequences, hydrogenase classes, taxonomic affiliations, and predicted behavior (Figure S4). To enable easy exploration of the data set, the database also provides access to an interface for searching, filtering, and sorting the data, as well as the capacity to download the results in CSV or FASTA format. There are individual pages for the 38 hydrogenase classes defined here (Table 1), including descriptions of their physiological role, genetic organization, taxonomic distribution, and biochemical features. This is supplemented with a compendium of structural information about the hydrogenases, which is integrated with the Protein Databank (PDB), as well as a library of over 500 literature references (Figure S5).

## Conclusions

To summarize, HydDB is a definitive resource for hydrogenase classification and analysis. The classifier described here provides a reliable, efficient, and convenient tool for hydrogenase classification and functional prediction. HydDB also provides browsing tools for the rapid analysis and retrieval of hydrogenase sequences. Finally, the manually-curated repository of class descriptions, hydrogenase structures, and literature references provides a deep but accessible resource for understanding hydrogenases.

## Methods

### Sequence datasets

The database was constructed using the amino acid sequences of all curated non-redundant 3248 hydrogenase catalytic subunits represented in the NCBI RefSeq database in August 2014^2^ (Dataset S1). In order to test the classification tool, additional sequences from newly-sequenced archaeal and bacterial phyla were retrieved from the Joint Genome Institute’s Integrated Microbial Genomes database^43^.

### Sequence similarity networks

Sequence similarity networks (SSNs)^18^ constructed using Cytoscape 4.1^44^ were used to visualize the distribution and diversity of the retrieved hydrogenase sequences. In this analysis, each node represents one of the 3248 hydrogenase sequences in the reference database (Dataset S1). Each edge represents the sequence similarity between them as determined by *E*-values from all-vs-all BLAST analysis, with all self and duplicate edges removed. Three networks were constructed, namely for the [NiFe]-hydrogenase large subunit sequences (Dataset S2), [FeFe]-hydrogenase catalytic domain sequences (Dataset S3), and [Fe]-hydrogenase sequences (Dataset S4). To control the degree of separation between nodes, log*E* cutoffs that were incrementally decreased from −5 to −200 until no major changes in clustering was observed. The log*E* cutoffs used for the final classifications are shown in Fig. 1 and Figure S1.

### Classification method

The 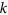-NN method is a well-known machine learning method for classification^45^. Given a set of data points *x*_*1*_, *x*_*2*_, … *x*_*N*_ (e.g. sequences) with known labels *y*_*1*_, *y*_*2*_, …, *y*_*N*_ (e.g. type annotations), the label of a point, 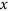, is predicted by computing the distance from 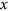 to *x*_*1*_, *x*_*2*_, … *x*_*N*_ and extracting the 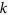 labeled points closest to 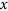, i.e. the neighbors. The predicted label is then determined by majority vote of the labels of the neighbors. The distance measure applied here is that of a BLAST search. Thus, the classifier corresponds to a homology search where the types of the top 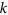 results are considered. However, formulating the classification method as a machine learning problem allows the use of common evaluation methods to estimate the precision of the method and perform model selection. The classifier was evaluated using 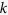-fold cross-validation. The dataset is first split in to 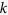 parts of equal size. 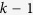 parts (the *training set*) are then used for training the classifier and the labels of the data points in the remaining part (the *test set*) are then predicted. This process, called a *fold*, is repeated 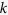 times. The predicted labels of each fold are then compared to the known labels and a precision can be computed.

## Additional Information

**How to cite this article**: Søndergaard, D. *et al*. HydDB: A web tool for hydrogenase classification and analysis. *Sci. Rep.* **6**, 34212; doi: 10.1038/srep34212 (2016).

## Supplementary Material

Supplementary Information

Supplementary Dataset S1

Supplementary Dataset S2

Supplementary Dataset S3

Supplementary Dataset S4

## Figures and Tables

**Table 1:** Expanded classification scheme for hydrogenase enzymes.

[NiFe] Group 1: Respiratory H_2_-uptake [NiFe]-hydrogenases			
1a	Periplasmic	Electron input for sulfate, metal, and organohalide respiration. [NiFeSe] variants.	2
1b	Prototypical	Electron input for sulfate, fumarate, metal, and nitrate respiration.	2
1c	Hyb-type	Electron input for fumarate, nitrate, and sulfate respiration. Physiologically reversible.	2
1d	Oxygen-tolerant	Electron input for aerobic respiration and oxygen-tolerant anaerobic respiration.	2
1e	Isp-type	Electron input primarily for sulfur respiration. Physiologically reversible.	2
1f	Oxygen-protecting	Unresolved role. May liberate electrons to reduce reactive oxygen species.	2
1g	Crenarchaeota-type	Electron input primarily for sulfur respiration.	2
1h	Actinobacteria-type	Electron input for aerobic respiration. Scavenges electrons from atmospheric H_2_.	2,46
1i	Coriobacteria-type (putative)	Undetermined role. May liberate electrons for anaerobic respiration.	This work
1j	Archaeoglobi-type	Electron input for sulfate respiration^π^.	This work
1k	Methanophenazine-reducing	Electron input for methanogenic heterodisulfide respiration^22^.	This work
[NiFe] Group 2: Alternative and sensory uptake [NiFe]-hydrogenases			
2a	Cyanobacteria-type	Electron input for aerobic respiration. Recycles H_2_ produced by other cellular processes.	16
2b	Histidine kinase-linked	H_2_ sensing. Activates two-component system controlling hydrogenase expression.	16
2c	Diguanylate cyclase-linked (putative)	Undetermined role. May sense H_2_ and regulate processes through cyclic di-GMP production.	2
2d	Aquificae-type	Unresolved role. May generate reductant for carbon fixation or have a regulatory role.	2
2e	Metallosphaera-type (putative)	Undetermined role. May liberate electrons primarily for aerobic respiration^26^.	This work
[NiFe] Group 3: Cofactor-coupled bidirectional [NiFe]-hydrogenases			
3a	F_420_-coupled	Couples oxidation of H_2_ to reduction of F_420_ during methanogenesis. Physiologically reversible. [NiFeSe] variants.	16
3b	NADP-coupled	Couples oxidation of NADPH to evolution of H_2_. Physiologically reversible. May have sulfhydrogenase activity.	16
3c	Heterodisulfide reductase-linked	Bifurcates electrons from H_2_ to heterodisulfide and Fd_ox_ in methanogens. [NiFeSe] variants.	16
3d	NAD-coupled	Interconverts electrons between H_2_ and NAD depending on cellular redox state.	16
[NiFe] Group 4: Respiratory H_2_-evolving [NiFe]-hydrogenases			
4a	Formate hydrogenlyase	Couples formate oxidation to fermentative H_2_ evolution. May be H^+^-translocating.	2
4b	Formate-respiring	Respires formate or carbon monoxide using H^+^ as electron acceptor. Na^+^-translocating via Mrp^23^.	This work
4c	Carbon monoxide-respiring	Respires carbon monoxide using H^+^ as electron acceptor. H^+^-translocating.	2
4d	Ferredoxin-coupled, Mrp-linked	Couples Fd_red_ oxidation to H^+^ reduction. Na^+^-translocating via Mrp complex^24^.	This work
4e	Ferredoxin-coupled, Ech-type	Couples Fd_red_ oxidation to H^+^ reduction. Physiologically reversible via H^+^/Na^+^ translocation.	2
4f	Formate-coupled (putative)	Undetermined role. May couple formate oxidation to H_2_ evolution and H^+^ translocation.	2
4g	Ferredoxin-coupled (putative)	Undetermined role. May couple Fd_red_ oxidation to proton reduction and H^+^/Na^+^ translocation.	This work
4h	Ferredoxin-coupled, Eha-type	Couples Fd_red_ oxidation to H^+^ reduction in anaplerotic processes. H^+^/Na^+^-translocating^25^.	This work
4i	Ferredoxin-coupled, Ehb-type	Couples Fd_red_ oxidation to H^+^ reduction in anabolic processes. H^+^/Na^+^-translocating^25^.	This work
[FeFe] Hydrogenases			
A1	Prototypical	Couples ferredoxin oxidation to fermentative or photobiological H_2_ evolution.	2,17
A2	Glutamate synthase-linked (putative)	Undetermined role. May couple H_2_ oxidation to NAD reduction, generating reductant for glutamate synthase.	2,17
A3	Bifurcating	Reversibly bifurcates electrons from H_2_ to NAD and Fd_ox_ in anaerobic bacteria.	2,17
A4	Formate dehydrogenase-linked	Couples formate oxidation to H_2_ evolution. Some bifurcate electrons from H_2_ to ferredoxin and NADP.	2,17
B	Colonic-type (putative)	Undetermined role. May couple Fd_red_ oxidation to fermentative H_2_ evolution.	17
C1	Histidine kinase-linked (putative)	Undetermined role. May sense H_2_ and regulate processes via histidine kinases^2^.	This work
C2	Chemotactic (putative)	Undetermined role. May sense H_2_ and regulate processes via methyl-accepting chemotaxis proteins^2^.	This work
C3	Phosphatase-linked (putative)	Undetermined role. May sense H_2_ and regulate processes via serine/threonine phosphatases^2^.	This work
[Fe] Hydrogenases			
All	Methenyl-H_4_MPT dehydrogenase	Reversibly couples H_2_ oxidation to 5,10-methenyltetrahydromethanopterin reduction.	16

The majority of the classes were defined in previous work^2^,^16^,^17^,^46^. The [NiFe] Group 1i, 1j, 2e, 4d, 4g, 4h, and 4i enzymes and [FeFe] Groups C1, C2, and C3 enzymes were defined in this work based on their separation into distinct clusters in the SSN analysis (Fig. 1). HydDB contains detailed information on each of these classes, including their taxonomic distribution, genetic organization, biochemistry, and structures, as well a list of primary references.

**Table 2:** Predictive capacity of the HydDB.

Organism	Phylum	Hydrogenase accession no.	HydDB classification	SSN classification	Predicted H_2_ metabolism	Confirmed H_2_ metabolism
Pyrinomonas methylaliphatogenes	Acidobacteria	WP_041979300.1	[NiFe] Group 1h	[NiFe] Group 1h	Persistence by aerobic respiration of atmospheric H_2_	Confirmed experimentally^31^
Phaeodactylibacter xiamenensis	Bacteroidetes	WP_044227713.1 WP_044216927.1 WP_044227053.1	[NiFe] Group 1d [NiFe] Group 2a [NiFe] Group 3d	[NiFe] Group 1d [NiFe] Group 2a [NiFe] Group 3d	Chemolithoautotrophic growth by aerobic H_2_ oxidation	Bacterium grows aerobically, but H_2_ oxidation untested^32^
Bathyarchaeota archaeon BA1	Bathyarchaeota	KPV62434.1 KPV62673.1 KPV62298.1	[NiFe] Group 3c [NiFe] Group 3c [NiFe] Group 4g	[NiFe] Group 3c [NiFe] Group 3c [NiFe] Group 4g	Couples Fd_red_ oxidation to H_2_ evolution in energy-conserving and bifurcating processes	Unconfirmed but consistent with metagenome-based models^36^
Lenisia limosa	Obazoa (Breviatea class)	LenisMan28	[FeFe] Group A1	[FeFe] Group A	Fermentative evolution of H_2_	Confirmed experimentally^47^
Acidianus copahuensis	Crenarchaeota	WP_048100721.1 WP_048100713.1 WP_048100378.1 WP_048100359.1	[NiFe] Group 1g [NiFe] Group 1g [NiFe] Group 1h [NiFe] Group 2e	[NiFe] Group 1g [NiFe] Group 1g [NiFe] Group 1h [NiFe] Group 2e	Chemolithoautotrophic growth by H_2_ oxidation using O_2_ or S_0_ as electron acceptors	Partially confirmed experimentally^27^
Arcobacter sp. E1/2/3	Proteobacteria (Epsilon class)	Arc.peg.2312	[NiFe] Group 1b	[NiFe] Group 1b	Chemolithoautotrophic growth by anaerobic H_2_ oxidation	Confirmed experimentally^47^
Methanoperedens nitroreducens	Euryarchaeota (ANME)	WP_048088262.1 WP_048090768.1	[NiFe] Group 3b [NiFe] Group 3b	[NiFe] Group 3b [NiFe] Group 3b	Secondary role for H_2_ metabolism limited to fermentative evolution of H_2_	Unconfirmed but consistent with metagenome-based models^35^
Kryptonium thompsoni	Kryptonia	CUU03002.1 CUU06124.1	[NiFe] Group 1d [NiFe] Group 3b	[NiFe] Group 1d [NiFe] Group 3b	Chemolithoautotrophic growth by aerobic H_2_ oxidation, fermentative evolution of H_2_.	Untested, candidate phylum identified by metagenomics^39^
Lokiarchaeum sp. GC14_75	Lokiarchaeota	KKK40681.1	[NiFe] Group 3c	[NiFe] Group 3c	Bifurcates electrons between H_2_, heterodisulfide, and ferredoxin	Unconfirmed but consistent with metagenome-based models^48^
Nitrospira moscoviensis	Nitrospirae	WP_053379275.1	[NiFe] Group 2a	[NiFe] Group 2a	Chemolithoautotrophic growth by aerobic H_2_ oxidation	Confirmed experimentally^33^
Bacterium GW2011_GWE1_35_17	Moranbacteria	KKQ46070.1 KKQ45273.1	[NiFe] Group 1a [NiFe] Group 3b	[NiFe] Group 1a [NiFe] Group 3b	Chemolithoautotrophic growth by anaerobic H_2_ oxidation, fermentative evolution of H_2_	Unconfirmed but consistent with metagenome-based models^37^
Bacterium GW2011_GWA2_33_10	Peregrinibacteria	KKP36897.1	[FeFe] Group A3	[FeFe] Group A	Bifurcates electrons between H_2_, NADH, and ferredoxin	Unconfirmed but consistent with metagenome-based models^37^
Entotheonella sp. TSY1	Tectomicrobia	ETW97737.1 ETW94065.1	[NiFe] Group 1h [NiFe] Group 3b	[NiFe] Group 1h [NiFe] Group 3b	Persistence by aerobic respiration of atmospheric H_2_, fermentative evolution of H_2_	Untested, candidate phylum identified by metagenomics^40^

HydDB accurately determined hydrogenase content and predicted the physiological roles of H_2_ metabolism in 12 newly-sequenced archaeal and bacterial species.
