# Supplementary material for: HydDB: A web tool for hydrogenase classification and analysis
Source: Sci Rep. 2016 Sep 27;6:34212. doi: 10.1038/srep34212 (PMC5037454; doi:10.1038/srep34212)
Supplement: Supplementary Information [file srep34212-s1.pdf]

HydDB: A web tool for hydrogenase classification and analysis

Dan Søndergaard[a], Christian N. S. Pedersen[a], Chris Greening[b, c*]

[a] Aarhus University, Bioinformatics Research Centre, C.F. Møllers Allé 8, Aarhus DK-8000, Denmark

[b] The Commonwealth Scientific and Industrial Research Organisation, Land and Water Flagship, Clunies Ross Street, Acton, ACT 2060, Australia

[c] Monash University, School of Biological Sciences, Clayton, VIC 2800, Australia

Correspondence:

Dr Chris Greening (chris.greening@monash.edu), Monash University, School of Biological Sciences, Clayton, VIC 2800, Australia

Dan Søndergaard (das@birc.au.dk), Aarhus University, Bioinformatics Research Centre, C.F. Møllers Allé 8, Aarhus DK-8000, Denmark

Figure S1. Sequence similarity networks showing the relationships between closely related subgroups of [NiFe]-hydrogenases as narrow log*E* filters.

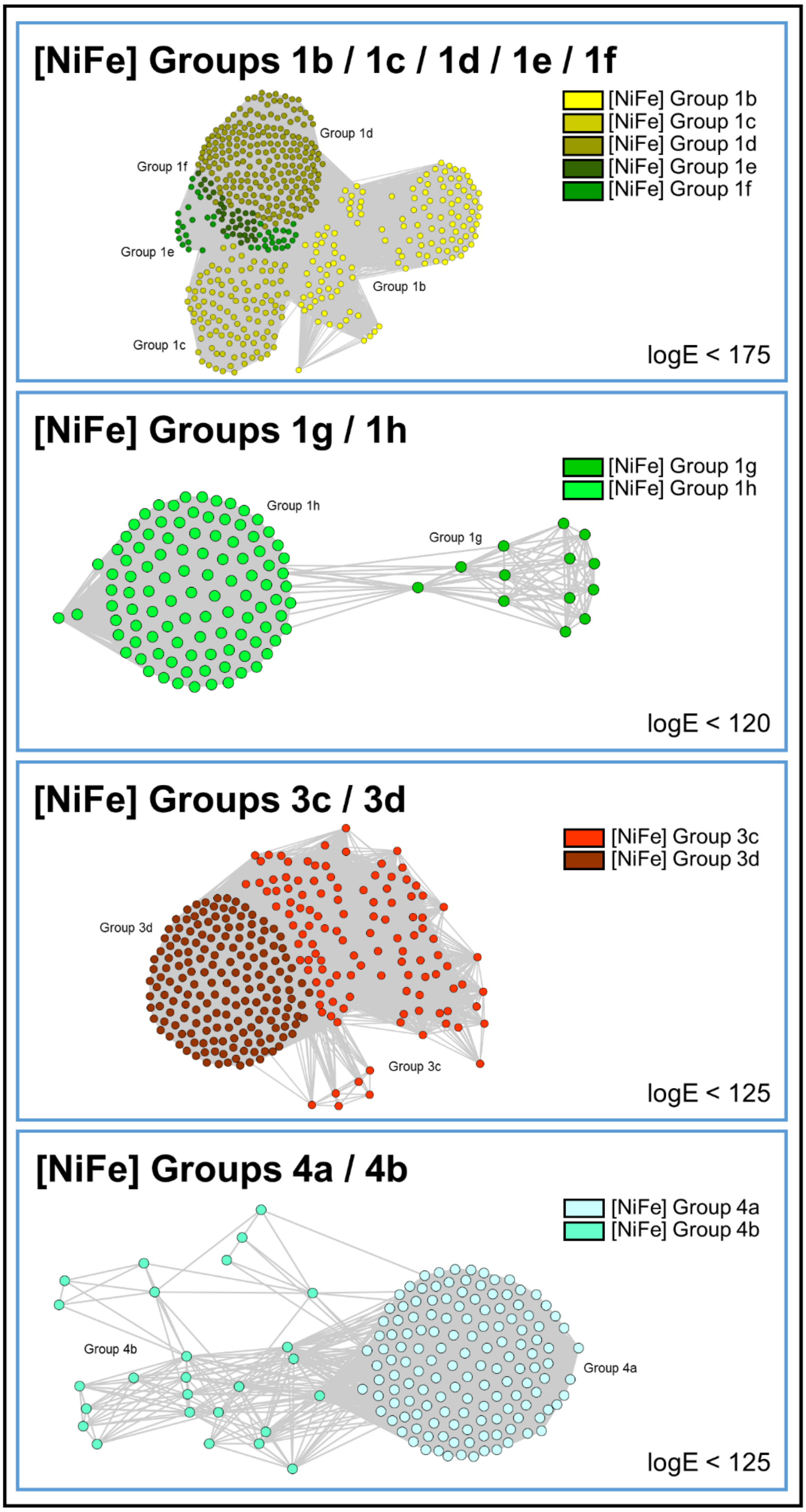

Figure S2. Screenshot showing interface of HydDB classification page.

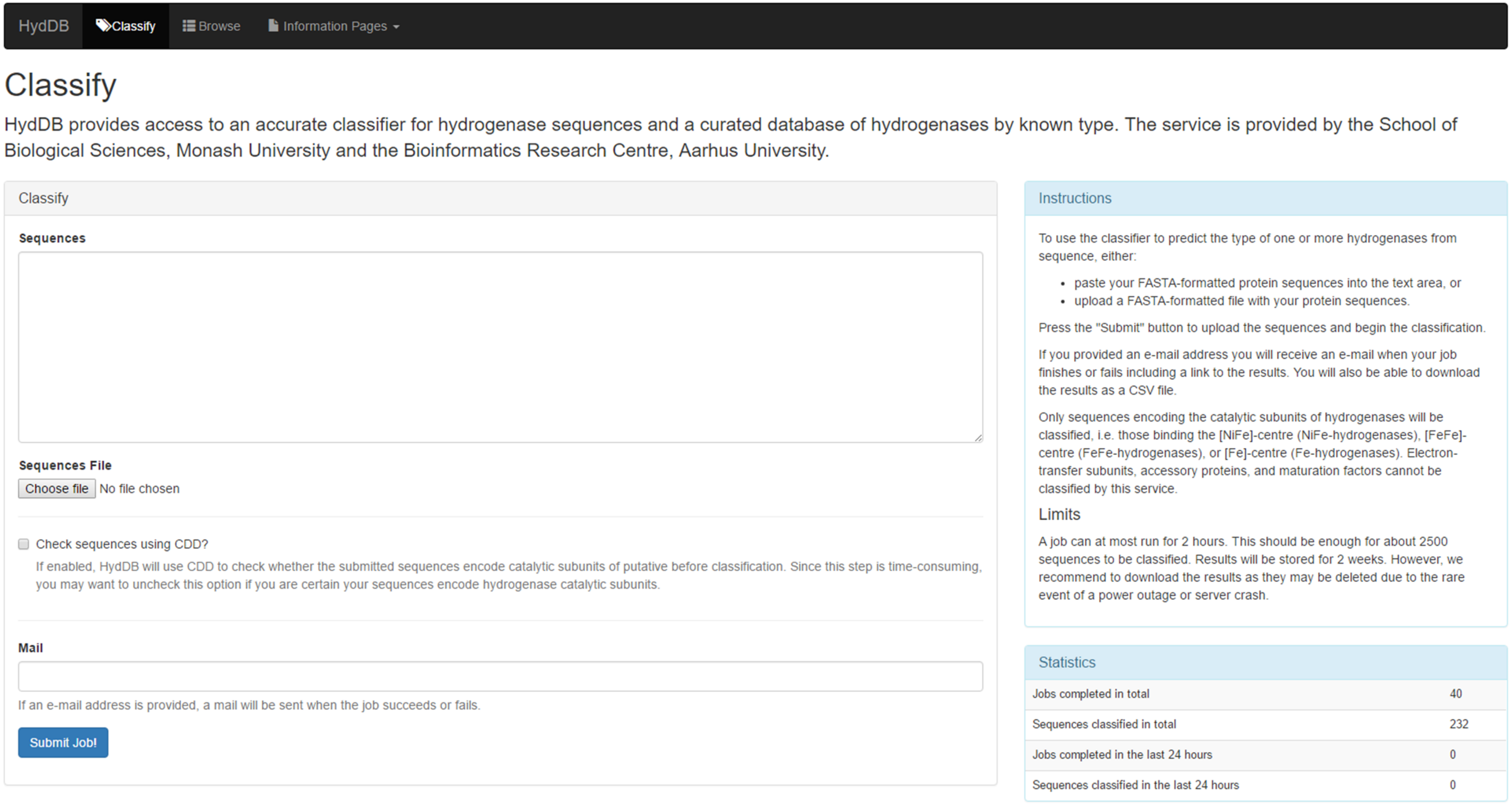

Figure S3. Screenshot showing the information provided in the data entry pages for 3248 individual hydrogenases in HydDB.

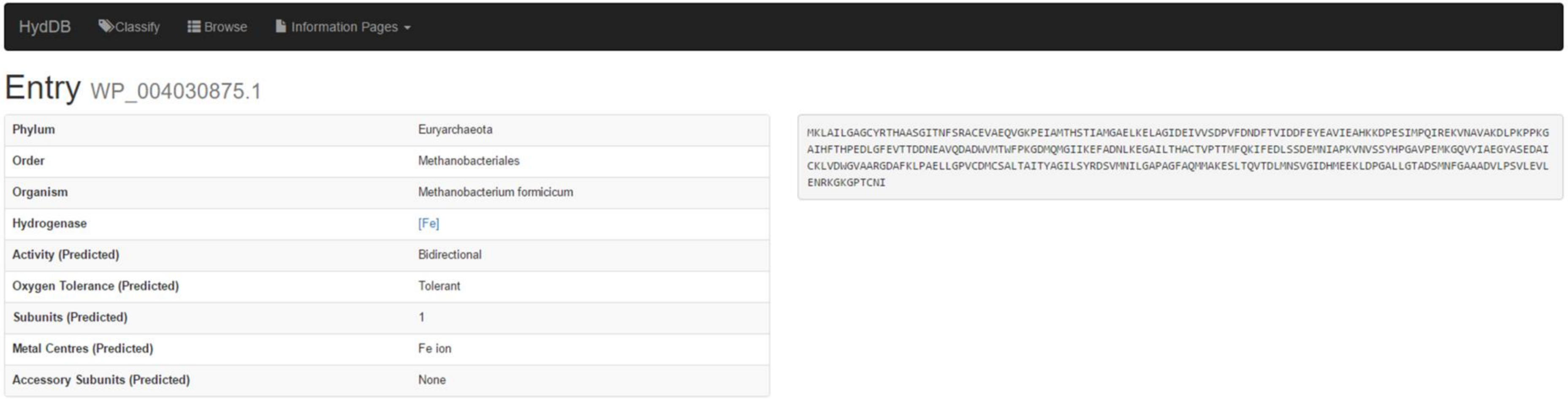

Figure S4. Screenshot showing the capacity for browsing hydrogenase data entries in HydDB.

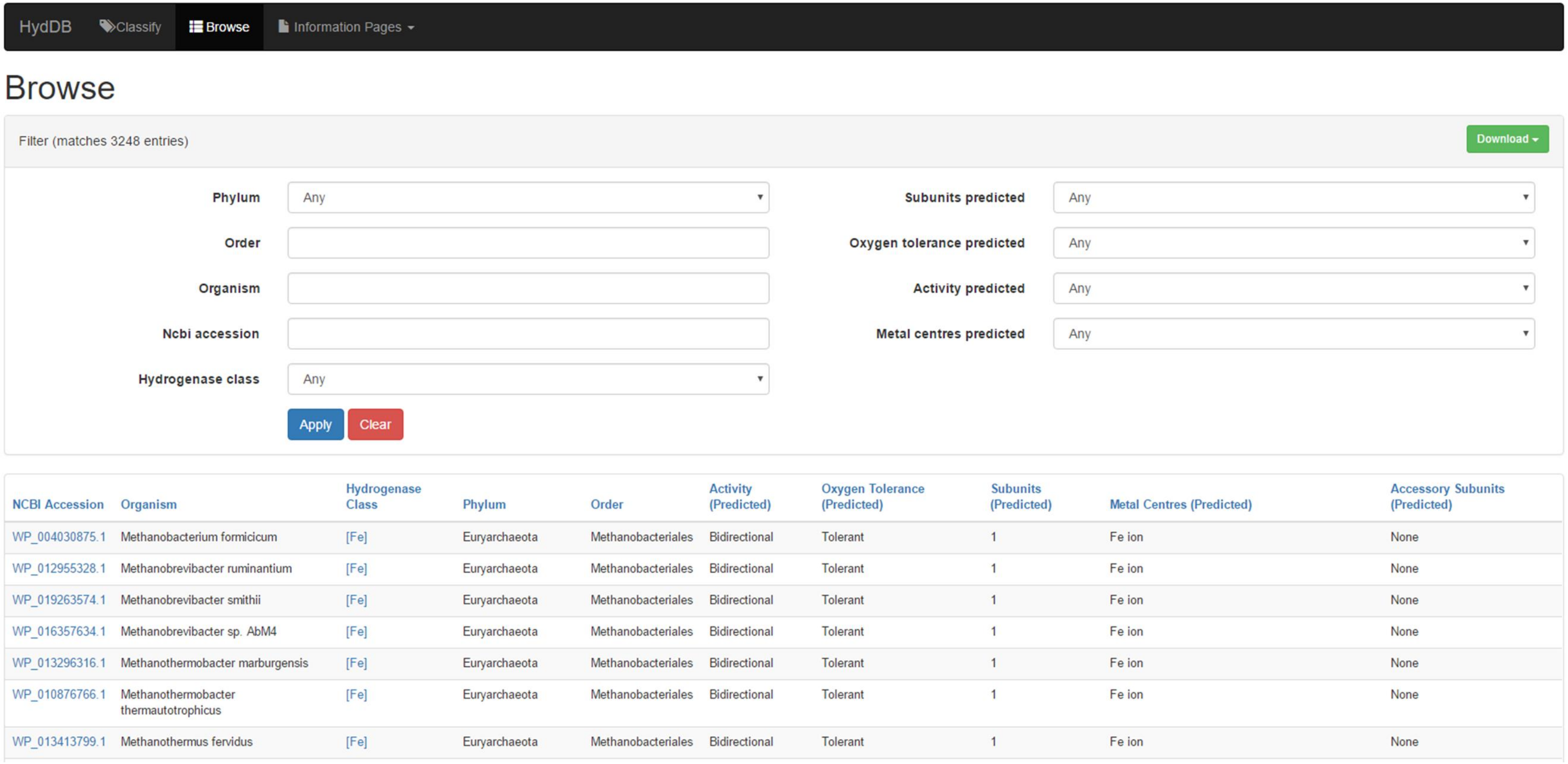

Figure S5. Screenshot showing the detailed content of the information pages about each hydrogenase class on HydDB. Equivalent information pages are available for all 38 hydrogenase classes defined in this work **(Table 1)**.

[NiFe] Group 1h-hydrogenase

This entry was last updated at: June 13, 2016, 11:11 a.m.

Properties

**Group**	[NiFe] Group 1: Respiratory H_2-uptake [NiFe] hydrogenases
**Subgroup**	[NiFe] Group 1h: Actinobacteria-type
**Function**	Hydrogenotrophic respiration using O_2 as terminal electron acceptor. Enzyme scavenges electrons from atmospheric H_2 to fuel respiratory chain during carbon-starvation. Route of electron transfer unresolved.
**Activity**	H_2-uptake (unidirectional, high-affinity)
**Oxygen tolerance**	O_2-tolerant or O_2-insensitive
**Localisation**	Membrane-associated?

Distribution

**Ecosystem distribution**	Upland soils, plant tissues, possibly surface waters
**Taxonomic distribution**	Widespread among obligately aerobic soil bacteria, especially Actinobacteria, Acidobacteria, and Chloroflexi

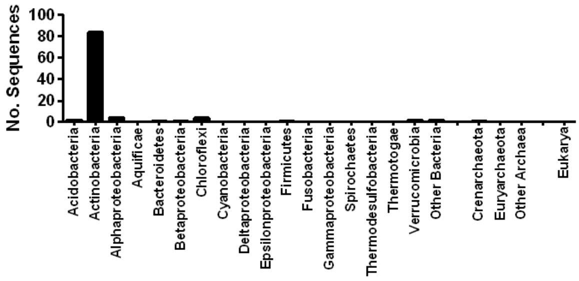

Genetic Organisation

e.g. *Mycobacterium smegmatis* (WP_003893634.1):

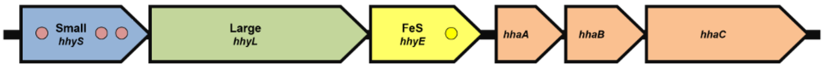

Architecture

**Structures**	5AA5 (*Ralstonia eutropha*, 2.5 Å resolution, active)
**Subunits**	3?
**Subunit description**	HyhL (hydrogenase large subunit)<br>HyhS (hydrogenase small subunit) HyhE (putative iron-sulfur protein and proposed physiological electron acceptor)
**Catalytic site**	[NiFe]-centre
**FeS clusters**	Proximal: 3Cys1Asp[4Fe4S]<br>Medial: 4Cys[4Fe4S]<br>Distal: 3Cys1His[4Fe4S]

Important Notes

The *Robiginitalea biformata* and *Sulfolobus islandicus* enzymes are relatively to distantly related to the main group. No studies have yet tested whether these enzymes have a H_2-scavenging role like other Group 1h [NiFe]-hydrogenases. They may instead represent founding members of a functionally-distinct lineage.

Sequences in this class

NCBI Accession	Organism	Hydrogenase Class	Phylum	Order	Activity (Predicted)	Oxygen Tolerance (Predicted)	Subunits (Predicted)	Metal Centres (Predicted)	Accessory Subunits (Predicted)
WP_014267363.1	Granulicella mallensis	[NiFe] Group 1h	Acidobacteria	Acidobacteriales	Aerobic Uptake	Tolerant	3	[NiFe]-centre, 3 x [4Fe4S] clusters	[FeS] protein
WP_011688202.1	Solibacter usitatus	[NiFe] Group 1h	Acidobacteria	Solibacterales	Aerobic Uptake	Tolerant	3	[NiFe]-centre, 3 x [4Fe4S] clusters	[FeS] protein
WP_021597135.1	Actinomadura madurae	[NiFe] Group 1h	Actinobacteria	Actinomycetales	Aerobic Uptake	Tolerant	3	[NiFe]-centre, 3 x [4Fe4S] clusters	[FeS] protein
WP_026402909.1	Actinomadura rifamycini	[NiFe] Group 1h	Actinobacteria	Actinomycetales	Aerobic Uptake	Tolerant	3	[NiFe]-centre, 3 x [4Fe4S] clusters	[FeS] protein
WP_018330638.1	Actinomycetospora chiangmaiensis	[NiFe] Group 1h	Actinobacteria	Actinomycetales	Aerobic Uptake	Tolerant	3	[NiFe]-centre, 3 x [4Fe4S] clusters	[FeS] protein
WP_007735075.1	Rhodococcus qingshengii	[NiFe] Group 1h	Actinobacteria	Actinomycetales	Aerobic Uptake	Tolerant	3	[NiFe]-centre, 3 x [4Fe4S] clusters	[FeS] protein
WP_003935326.1	Rhodococcus ruber	[NiFe] Group 1h	Actinobacteria	Actinomycetales	Aerobic Uptake	Tolerant	3	[NiFe]-centre, 3 x [4Fe4S] clusters	[FeS] protein
WP_005443931.1	Saccharomonospora azurea	[NiFe] Group 1h	Actinobacteria	Actinomycetales	Aerobic Uptake	Tolerant	3	[NiFe]-centre, 3 x [4Fe4S] clusters	[FeS] protein

« 1 2 3 »

Literature

Genetics:

- Berney, M., Greening, C., Hards, K., Collins, D., and Cook, G.M. (2014) Three different [NiFe] hydrogenases confer metabolic flexibility in the obligate aerobe *Mycobacterium smegmatis*. *Environ. Microbiol.* **16**: 318-330.
- Constant, P., Chowdhury, S.P., Hesse, L., and Conrad, R. (2011) Co-localization of atmospheric H_2 oxidation activity and high affinity H_2-oxidizing bacteria in non-axenic soil and sterile soil amended with *Streptomyces* sp. PCB7. *Soil Biol. Biochem.* **43**: 1888-1893.
- Constant, P., Chowdhury, S.P., Hesse, L., Pratscher, J., and Conrad, R. (2011) Genome data mining and soil survey for the novel group 5 [NiFe]-hydrogenase to explore the diversity and ecological importance of presumptive high-affinity H_2-oxidizing bacteria. *Appl. Environ. Microbiol.* **77**: 6027-6035.
- Greening, C., Biswas, A., Carere, C.R., Jackson, C.J., Taylor, M.C., Stott, M.B., Cook, G.M., and Morales, S.E. (2016) Genomic and metagenomic surveys of hydrogenase distribution indicate H_2 is a widely utilised energy source for microbial growth and survival. *ISME J.* **10**: 761-777.
- Khdhiri, M., Hesse, L., Popa, M.E., Quiza, L., Lalonde, I., Meredith, L.K., Röckmann, T., and Constant, P. (2015) Soil carbon content and relative abundance of high affinity H_2-oxidizing bacteria predict atmospheric H_2 soil uptake activity better than soil microbial community composition. *Soil Biol. Biochem.* **85**: 1-9.

Physiology:

- Berney, M., Greening, C., Conrad, R., Jacobs, W.R., and Cook, G.M. (2014) An obligately aerobic soil bacterium activates fermentative hydrogen production to survive reductive stress during hypoxia. *Proc. Natl. Acad. Sci. U. S. A.* **111**: 11479-11484.
- Constant, P., Chowdhury, S.P., Pratscher, J., and Conrad, R. (2010) Streptomycetes contributing to atmospheric molecular hydrogen soil uptake are widespread and encode a putative high-affinity [NiFe]-hydrogenase. *Environ. Microbiol.* **12**: 821-829.

Table S1. Validation that HydDB classifies only hydrogenase catalytic subunit sequences. HydDB excludes non-hydrogenase sequences through a combination of homology checks (sequences are only classified as hydrogenases if BLAST *E*-value of the closest hit in HydDB is less than 10^{-5}) and CDD checks (sequences are only classified as hydrogenases if signature conserved domains are found). In addition, the classifier has been specifically trained to exclude four protein families that are homologous to hydrogenase catalytic subunits (HmdII, Her, NuoD, NARF) but lack hydrogenase activity.

**NCBI Accession**	**Sequence type**	**Homology check**	**CDD check**	**Final result**
WP_041979300.1	Validated hydrogenase catalytic subunit	Highest sequence homology with [NiFe] Group 1h (E = 0)	Ni,Fe-hydrogenase I large subunit (COG0374)	Hydrogenase
WP_011729412.1	P-type ATPase (unrelated to hydrogenases)	Low sequence homology with hydrogenases (E = 5.6)	Non-hydrogenase	Non-hydrogenase
WP_003895387.1	Chaperone (unrelated to hydrogenases)	Low sequence homology with hydrogenases (E = 3.8)	Non-hydrogenase	Non-hydrogenase
WP_013295714.1	HmdII (homologous with [Fe]-hydrogenases)	Highest sequence homology with HmdII (E = 0)	HMD (pfam03201)	Non-hydrogenase
WP_003901794.1	Ehr (homologous with [NiFe]-hydrogenases)	Highest sequence homology with Ehr (E = 0)	Complex1_49kDa superfamily (cl21493)	Non-hydrogenase
WP_003901553.1	NuoD (homologous with [NiFe]-hydrogenases)	Highest sequence homology with NuoD (E = 0)	NuoD (COG0649)	Non-hydrogenase
NP_114174.1	NARF (homologous with [FeFe]-hydrogenases)	Highest sequence homology with NARF (E = 0)	Fe_hyd_Ig_C (pfam02906)	Non-hydrogenase

Table S2. Hydrogenase sequences where there is disagreement between classification by SSN and *k*-NN methods. These sequences represent six out of the total 3248 sequences analyzed, i.e. 0.0018%.

**NCBI Accession**	**Organism**	***k*-NN Classification**	**SSN Classification**
WP_027414715.1	Aneurinibacillus terranovensis	[NiFe] Group 1e	[NiFe] Group 1d
WP_027358538.1	Desulforegula conservatrix	[NiFe] Group 3d	[NiFe] Group 3c
WP_012532312.1	Geobacter bemidjiensis	[NiFe] Group 3d	[NiFe] Group 3c
WP_012469611.1	Geobacter lovleyi	[NiFe] Group 3d	[NiFe] Group 3c
WP_004512544.1	Geobacter metallireducens	[NiFe] Group 3d	[NiFe] Group 3c
WP_015839165.1	Geobacter sp. M21	[NiFe] Group 3d	[NiFe] Group 3c

Dataset S1. Excel spreadsheet listing the sequence, taxonomy, and hydrogenase class of all 3248 hydrogenase catalytic subunit sequences listed in HydDB.

Dataset S2. Zip file containing the Cytoscape network for [NiFe]-hydrogenases.

Dataset S3. Zip file containing the Cytoscape network for [FeFe]-hydrogenases.

Dataset S4. Zip file containing the Cytoscape network for [Fe]-hydrogenases.
